# 396. Clinical Characterization of Long COVID in Mexico

**DOI:** 10.1093/ofid/ofad500.466

**Published:** 2023-11-27

**Authors:** Luis Del Carpio-Orantes, Sergio García-Mendez, Jesús Salvador Sánchez-Díaz, Andrés Aguilar-Silva, Manuel Martínez-Rojas, Oscar Rodrigo Jiménez-Flores, Luis Roberto Villalobos-López, América Alejandrina González-Arce, Karem Samantha González-Medel, Rubén Domínguez-Cámara, Alvaro Efrén Munguía-Sereno, Violeta Rosalia Zamora-Vázquez, Quiahuitzin Elizabeth Vázquez-Manzano, Diego Ortíz-Pérez, Semiramis Itzel Hernández-Martínez, Sara Nohemí Hernández-Hernández, Ishar Solís-Sánchez, Victor Alejandro Fonseca-Pouchoulen, Laura Guadalupe Montano-Montiel, Reynaldo Reich-Sierra

**Affiliations:** Grupo de Estudio para el Diagnóstico y Tratamiento de COVID-19 en Veracruz, México, Veracruz, Veracruz-Llave, Mexico; Study Group for the Diagnosis and Treatment of COVID-19 in Veracruz, Mexico, Veracruz, Veracruz-Llave, Mexico; Instituto Mexicano del Seguro Social, Veracruz, Veracruz-Llave, Mexico; Instituto Mexicano del Seguro Social, Veracruz, Veracruz-Llave, Mexico; Instituto Mexicano del Seguro Social, Veracruz, Veracruz-Llave, Mexico; Instituto Mexicano del Seguro Social, Veracruz, Veracruz-Llave, Mexico; Instituto Mexicano del Seguro Social, Veracruz, Veracruz-Llave, Mexico; Instituto Mexicano del Seguro Social, Veracruz, Veracruz-Llave, Mexico; Study Group for the Diagnosis and Treatment of COVID-19, Veracruz, Veracruz-Llave, Mexico; Instituto Mexicano del Seguro Social, Veracruz, Veracruz-Llave, Mexico; Study Group for the Diagnosis and Treatment of COVID-19, Veracruz, Veracruz-Llave, Mexico; Study Group for the Diagnosis and Treatment of COVID-19 in Veracruz, Mexico, Veracruz, Veracruz-Llave, Mexico; Study Group for the Diagnosis and Treatment of COVID-19, Veracruz, Veracruz-Llave, Mexico; Study Group for the Diagnosis and Treatment of COVID-19 in Veracruz, Mexico, Veracruz, Veracruz-Llave, Mexico; Study Group for the Diagnosis and Treatment of COVID-19, Veracruz, Veracruz-Llave, Mexico; Study Group for the Diagnosis and Treatment of COVID-19 in Veracruz, Mexico, Veracruz, Veracruz-Llave, Mexico; Study Group for the Diagnosis and Treatment of COVID-19, Veracruz, Veracruz-Llave, Mexico; Study Group for the Diagnosis and Treatment of COVID-19 in Veracruz, Mexico, Veracruz, Veracruz-Llave, Mexico; Study Group for the Diagnosis and Treatment of COVID-19, Veracruz, Veracruz-Llave, Mexico; Study Group for the Diagnosis and Treatment of COVID-19 in Veracruz, Mexico, Veracruz, Veracruz-Llave, Mexico

## Abstract

**Background:**

Long COVID is defined as the persistence of symptoms after 4 weeks of an acute picture. There is talk of 65 million people affected in the world. In Mexico there are no statistics or studies that explore this entity in the population.

**Methods:**

A descriptive, cross-sectional, and prospective study was carried out using an online survey, in adults who wish to participate and who are living in Mexico from January to March 2023, whose main objective is to characterize patients who present symptoms of Long COVID

**Results:**

336 participants with an average age of 41 years (range 18-79 years), the most affected gender is female (69%); Risk factors are Obesity 41%, Diabetes 16%, Hypertension 15.8%; 43.5% commented that they were healthy before COVID-19. Cases of acute COVID they have suffered, 42.3% comment that 2 previous infections, 29.5% had one and 28.3% 3 or more. 77% refer to mild COVID, 13% to severe COVID, and 10% both. Regarding vaccination, 45% have 3 or more vaccines, 36% 2 vaccines, 9% have one vaccine and 10% have not been vaccinated against COVID-19. The most prevalent symptoms are Neuropsychiatric 90%, Musculoskeletal 88%, Cardiovascular 82%, Gastrointestinal 78%, and Pulmonary 71%. The most frequent Neuropsychiatric symptoms: fatigue 76%, memory disorder 72%, anxiety 65%; Musculoskeletal symptoms are arthralgia 71.4%, myalgia 40%, arthritis 28%. Cardiovascular symptoms are palpitations 58%, tachycardia 38%, precordial pain 27%. Gastrointestinal symptoms are diarrhea 43%, abdominal pain 41%, Colitis 26%. Pulmonary symptoms are chronic cough 40%, persistent expectoration 29%, dyspnea 23%. Other symptoms are alopecia 53.3%, chronic dermatitis 38%, frequent infections after COVID-19 20%, menstrual disorders 17%, thyroid disease 12%, development of autoimmune diseases 9.5%, sexual dysfunction 9%, COVID tests persistently positive 6%, thrombotic events 3.6% (cerebral 0.9%, pulmonary thromboembolism 0.6%, myocardial infarction 0.3%), myocarditis 3.3%, chronic renal failure 2.4% and cancer 1.8%.

**Figure:**
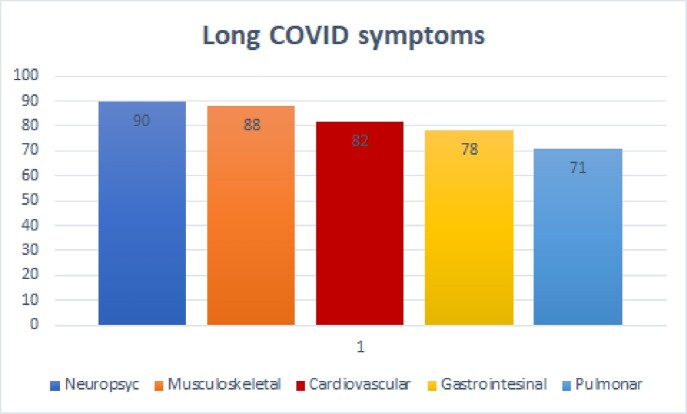

**Figure:**
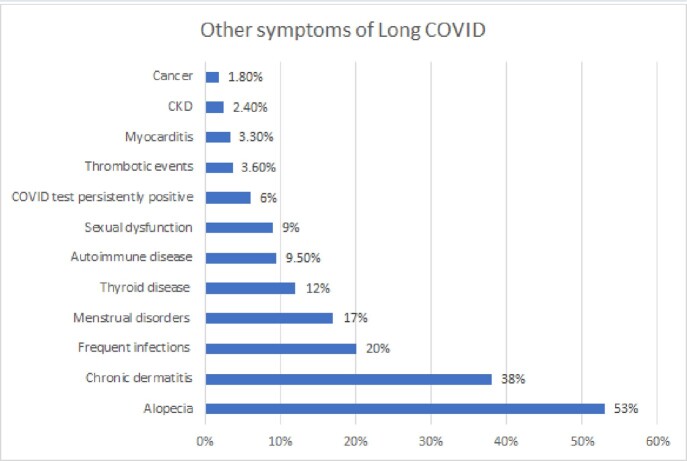

**Conclusion:**

It is important to characterize this population since it has particularities that make it susceptible to this new entity and the creation of clinical guidelines in the country should be encouraged to begin limiting sequelae.

**Disclosures:**

**All Authors**: No reported disclosures

